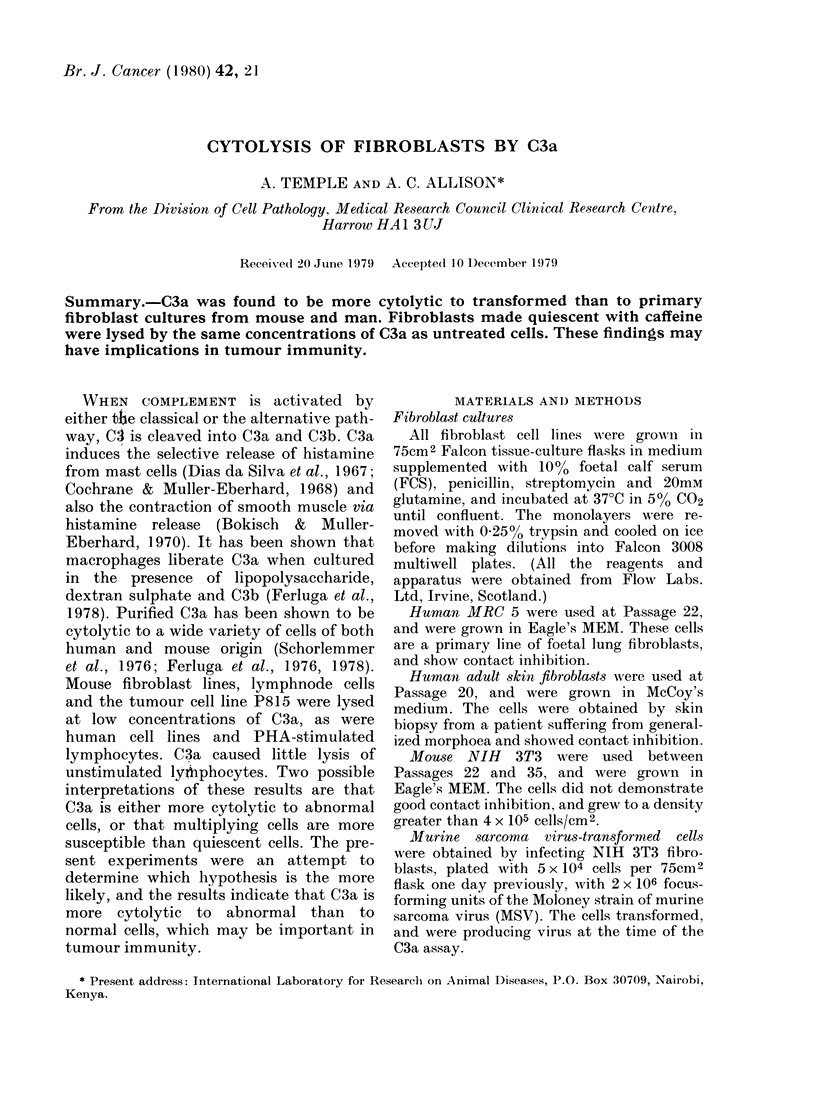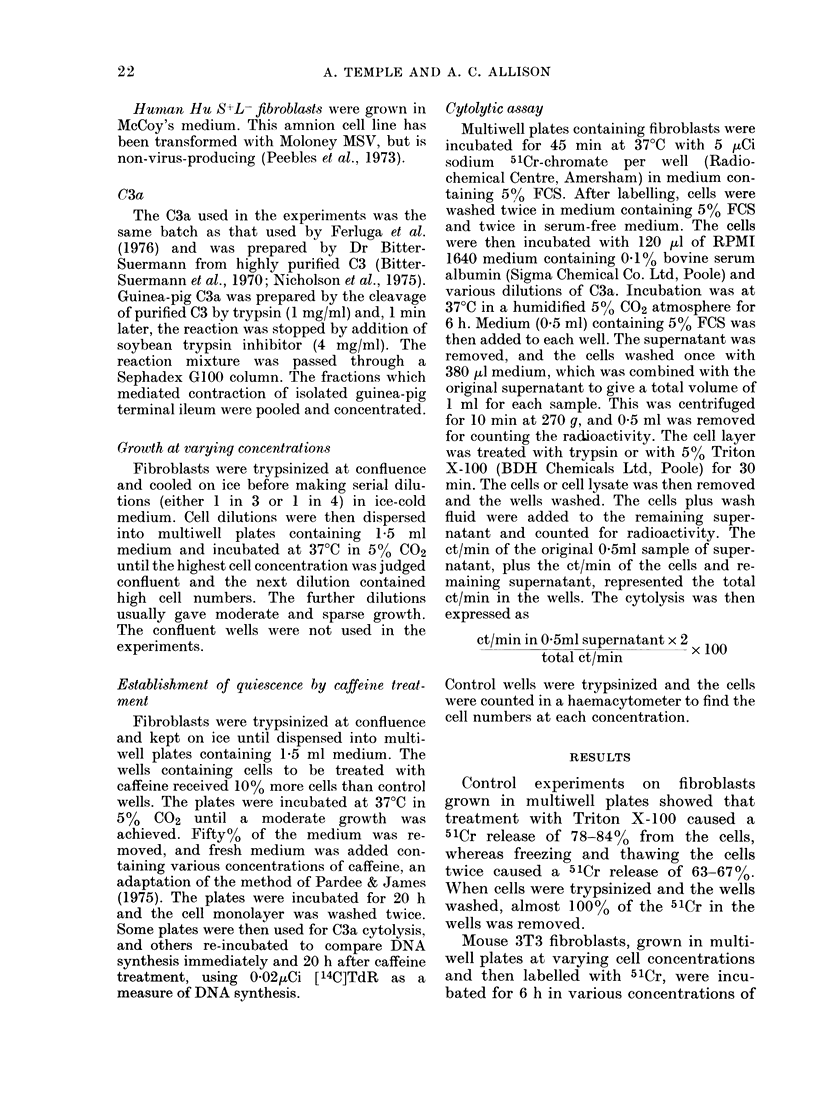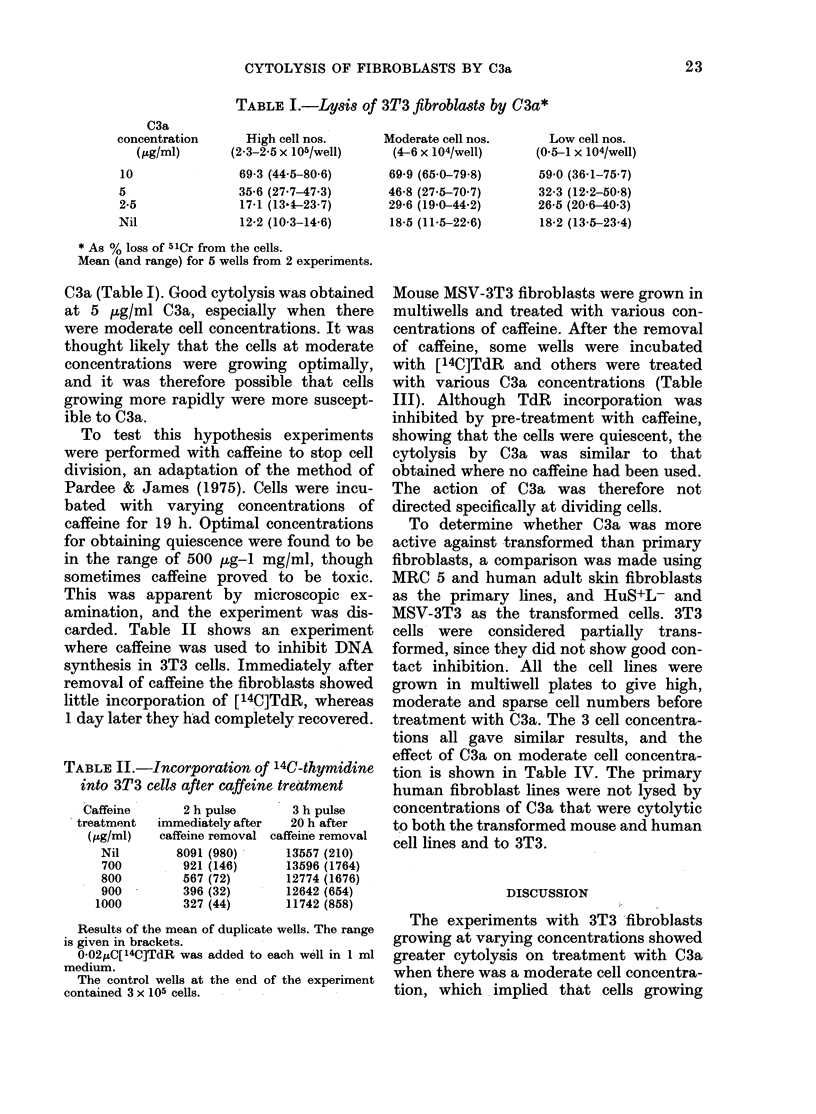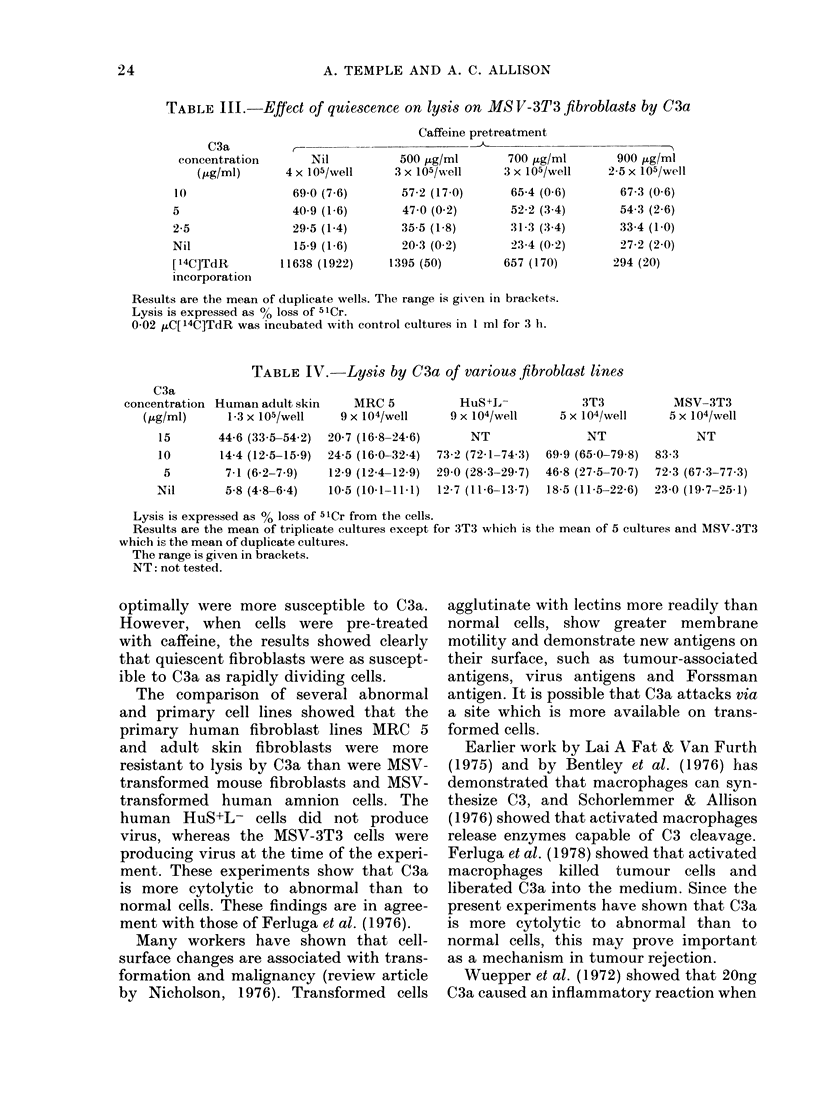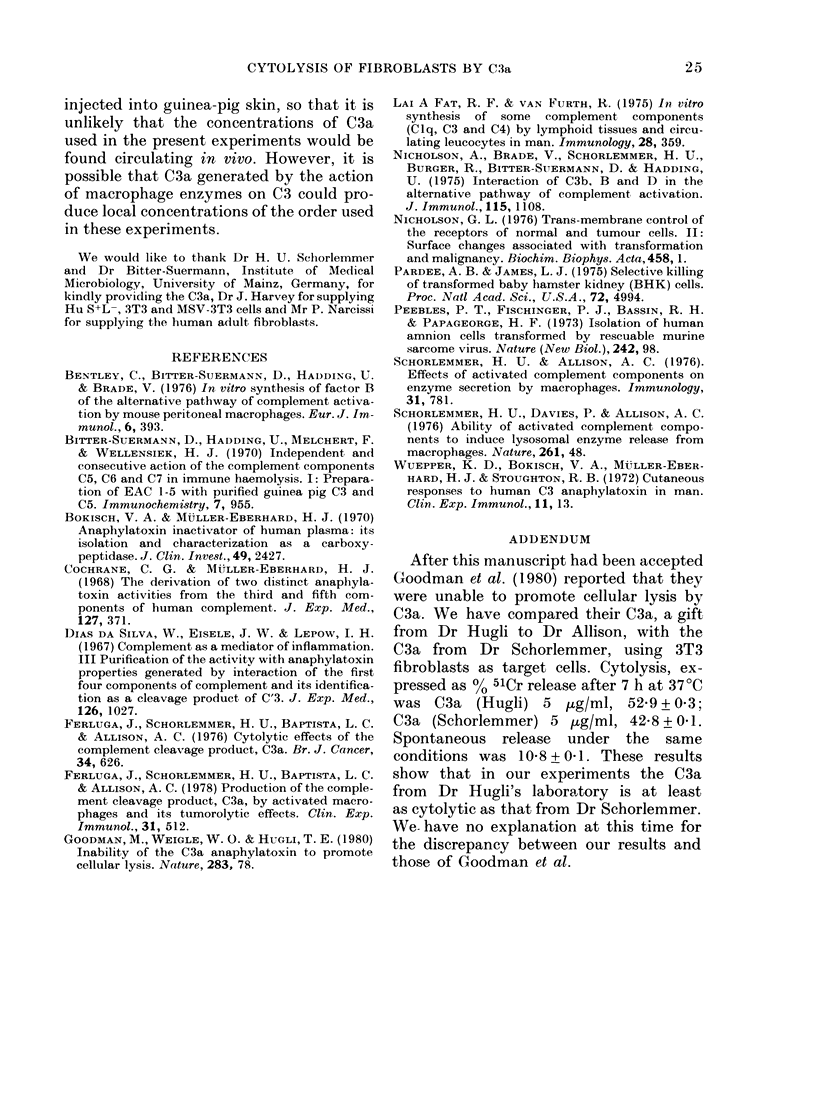# Cytolysis of fibroblasts by C3a.

**DOI:** 10.1038/bjc.1980.198

**Published:** 1980-07

**Authors:** A. Temple, A. C. Allison

## Abstract

C3a was found to be more cytolytic to transformed than to primary fibroblast cultures from mouse and man. Fibroblasts made quiescent with caffeine were lysed by the same concentrations of C3a as untreated cells. These findings may have implications in tumour immunity.


*Br. J. Cancer* (1980) **42**, 21

### CYTOLYSIS OF FIBROBLASTS BY C3a

A. TEMPLE AND A. C. ALLISON*

*From the Division of Cell Pathology, Medical Research Council Clinical Research Centre, Harrow HA1 3UJ*

Received 20 June 1979 Accepted 10 December 1979

**Summary.—C3a was found to be more cytolytic to transformed than to primary fibroblast cultures from mouse and man. Fibroblasts made quiescent with caffeine were lysed by the same concentrations of C3a as untreated cells. These findings may have implications in tumour immunity.**

WHEN COMPLEMENT is activated by either the classical or the alternative pathway, C3 is cleaved into C3a and C3b. C3a induces the selective release of histamine from mast cells (Dias da Silva *et al.*, 1967; Cochrane & Muller-Eberhard, 1968) and also the contraction of smooth muscle *via* histamine release (Bokisch & Muller-Eberhard, 1970). It has been shown that macrophages liberate C3a when cultured in the presence of lipopolysaccharide, dextran sulphate and C3b (Ferluga *et al.*, 1978). Purified C3a has been shown to be cytolytic to a wide variety of cells of both human and mouse origin (Schorlemmer *et al.*, 1976; Ferluga *et al.*, 1976, 1978). Mouse fibroblast lines, lymphnode cells and the tumour cell line P815 were lysed at low concentrations of C3a, as were human cell lines and PHA-stimulated lymphocytes. C3a caused little lysis of unstimulated lymphocytes. Two possible interpretations of these results are that C3a is either more cytolytic to abnormal cells, or that multiplying cells are more susceptible than quiescent cells. The present experiments were an attempt to determine which hypothesis is the more likely, and the results indicate that C3a is more cytolytic to abnormal than to normal cells, which may be important in tumour immunity.

### MATERIALS AND METHODS

### *Fibroblast cultures*

All fibroblast cell lines were grown in 75cm$^2$ Falcon tissue-culture flasks in medium supplemented with 10% foetal calf serum (FCS), penicillin, streptomycin and 20mM glutamine, and incubated at 37°C in 5% $CO_2$ until confluent. The monolayers were removed with 0·25% trypsin and cooled on ice before making dilutions into Falcon 3008 multiwell plates. (All the reagents and apparatus were obtained from Flow Labs. Ltd, Irvine, Scotland.)

*Human MRC* 5 were used at Passage 22, and were grown in Eagle's MEM. These cells are a primary line of foetal lung fibroblasts, and show contact inhibition.

*Human adult skin fibroblasts* were used at Passage 20, and were grown in McCoy's medium. The cells were obtained by skin biopsy from a patient suffering from generalized morphoea and showed contact inhibition.

*Mouse NIH* 3*T*3 were used between Passages 22 and 35, and were grown in Eagle's MEM. The cells did not demonstrate good contact inhibition, and grew to a density greater than $4 \times 10^5$ cells/cm$^2$.

*Murine sarcoma virus-transformed cells* were obtained by infecting NIH 3T3 fibroblasts, plated with $5 \times 10^4$ cells per 75cm$^2$ flask one day previously, with $2 \times 10^6$ focus-forming units of the Moloney strain of murine sarcoma virus (MSV). The cells transformed, and were producing virus at the time of the C3a assay.

* Present address: International Laboratory for Research on Animal Diseases, P.O. Box 30709, Nairobi, Kenya.

*Human Hu $S^+L^-$ fibroblasts* were grown in McCoy's medium. This amnion cell line has been transformed with Moloney MSV, but is non-virus-producing (Peebles *et al.*, 1973).

*C3a*

The C3a used in the experiments was the same batch as that used by Ferluga *et al.* (1976) and was prepared by Dr Bitter-Suermann from highly purified C3 (Bitter-Suermann *et al.*, 1970; Nicholson *et al.*, 1975). Guinea-pig C3a was prepared by the cleavage of purified C3 by trypsin (1 mg/ml) and, 1 min later, the reaction was stopped by addition of soybean trypsin inhibitor (4 mg/ml). The reaction mixture was passed through a Sephadex G100 column. The fractions which mediated contraction of isolated guinea-pig terminal ileum were pooled and concentrated.

*Growth at varying concentrations*

Fibroblasts were trypsinized at confluence and cooled on ice before making serial dilutions (either 1 in 3 or 1 in 4) in ice-cold medium. Cell dilutions were then dispersed into multiwell plates containing 1·5 ml medium and incubated at 37°C in 5% $CO_2$ until the highest cell concentration was judged confluent and the next dilution contained high cell numbers. The further dilutions usually gave moderate and sparse growth. The confluent wells were not used in the experiments.

*Establishment of quiescence by caffeine treatment*

Fibroblasts were trypsinized at confluence and kept on ice until dispensed into multiwell plates containing 1·5 ml medium. The wells containing cells to be treated with caffeine received 10% more cells than control wells. The plates were incubated at 37°C in 5% $CO_2$ until a moderate growth was achieved. Fifty% of the medium was removed, and fresh medium was added containing various concentrations of caffeine, an adaptation of the method of Pardee & James (1975). The plates were incubated for 20 h and the cell monolayer was washed twice. Some plates were then used for C3a cytolysis, and others re-incubated to compare DNA synthesis immediately and 20 h after caffeine treatment, using 0·02$\mu$Ci [$^{14}$C]TdR as a measure of DNA synthesis.

*Cytolytic assay*

Multiwell plates containing fibroblasts were incubated for 45 min at 37°C with 5 $\mu$Ci sodium $^{51}$Cr-chromate per well (Radiochemical Centre, Amersham) in medium containing 5% FCS. After labelling, cells were washed twice in medium containing 5% FCS and twice in serum-free medium. The cells were then incubated with 120 $\mu$l of RPMI 1640 medium containing 0·1% bovine serum albumin (Sigma Chemical Co. Ltd, Poole) and various dilutions of C3a. Incubation was at 37°C in a humidified 5% $CO_2$ atmosphere for 6 h. Medium (0·5 ml) containing 5% FCS was then added to each well. The supernatant was removed, and the cells washed once with 380 $\mu$l medium, which was combined with the original supernatant to give a total volume of 1 ml for each sample. This was centrifuged for 10 min at 270 $g$, and 0·5 ml was removed for counting the radioactivity. The cell layer was treated with trypsin or with 5% Triton X-100 (BDH Chemicals Ltd, Poole) for 30 min. The cells or cell lysate was then removed and the wells washed. The cells plus wash fluid were added to the remaining supernatant and counted for radioactivity. The ct/min of the original 0·5ml sample of supernatant, plus the ct/min of the cells and remaining supernatant, represented the total ct/min in the wells. The cytolysis was then expressed as

$$\frac{\text{ct/min in 0·5ml supernatant} \times 2}{\text{total ct/min}} \times 100$$

Control wells were trypsinized and the cells were counted in a haemacytometer to find the cell numbers at each concentration.

### RESULTS

Control experiments on fibroblasts grown in multiwell plates showed that treatment with Triton X-100 caused a $^{51}$Cr release of 78–84% from the cells, whereas freezing and thawing the cells twice caused a $^{51}$Cr release of 63–67%. When cells were trypsinized and the wells washed, almost 100% of the $^{51}$Cr in the wells was removed.

Mouse 3T3 fibroblasts, grown in multiwell plates at varying cell concentrations and then labelled with $^{51}$Cr, were incubated for 6 h in various concentrations of

TABLE I.—*Lysis of 3T3 fibroblasts by C3a**

| C3a concentration ($\mu$g/ml) | High cell nos. (2·3–2·5 × $10^5$/well) | Moderate cell nos. (4–6 × $10^4$/well) | Low cell nos. (0·5–1 × $10^4$/well) |
|---|---|---|---|
| 10 | 69·3 (44·5–80·6) | 69·9 (65·0–79·8) | 59·0 (36·1–75·7) |
| 5 | 35·6 (27·7–47·3) | 46·8 (27·5–70·7) | 32·3 (12·2–50·8) |
| 2·5 | 17·1 (13·4–23·7) | 29·6 (19·0–44·2) | 26·5 (20·6–40·3) |
| Nil | 12·2 (10·3–14·6) | 18·5 (11·5–22·6) | 18·2 (13·5–23·4) |

* As % loss of $^{51}$Cr from the cells.
Mean (and range) for 5 wells from 2 experiments.

C3a (Table I). Good cytolysis was obtained at 5 $\mu$g/ml C3a, especially when there were moderate cell concentrations. It was thought likely that the cells at moderate concentrations were growing optimally, and it was therefore possible that cells growing more rapidly were more susceptible to C3a.

To test this hypothesis experiments were performed with caffeine to stop cell division, an adaptation of the method of Pardee & James (1975). Cells were incubated with varying concentrations of caffeine for 19 h. Optimal concentrations for obtaining quiescence were found to be in the range of 500 $\mu$g–1 mg/ml, though sometimes caffeine proved to be toxic. This was apparent by microscopic examination, and the experiment was discarded. Table II shows an experiment where caffeine was used to inhibit DNA synthesis in 3T3 cells. Immediately after removal of caffeine the fibroblasts showed little incorporation of [$^{14}$C]TdR, whereas 1 day later they had completely recovered.

TABLE II.—*Incorporation of $^{14}$C-thymidine into 3T3 cells after caffeine treatment*

| Caffeine treatment ($\mu$g/ml) | 2 h pulse immediately after caffeine removal | 3 h pulse 20 h after caffeine removal |
|---|---|---|
| Nil | 8091 (980) | 13557 (210) |
| 700 | 921 (146) | 13596 (1764) |
| 800 | 567 (72) | 12774 (1676) |
| 900 | 396 (32) | 12642 (654) |
| 1000 | 327 (44) | 11742 (858) |

Results of the mean of duplicate wells. The range is given in brackets.
0·02$\mu$C[$^{14}$C]TdR was added to each well in 1 ml medium.
The control wells at the end of the experiment contained 3 × $10^5$ cells.

Mouse MSV-3T3 fibroblasts were grown in multiwells and treated with various concentrations of caffeine. After the removal of caffeine, some wells were incubated with [$^{14}$C]TdR and others were treated with various C3a concentrations (Table III). Although TdR incorporation was inhibited by pre-treatment with caffeine, showing that the cells were quiescent, the cytolysis by C3a was similar to that obtained where no caffeine had been used. The action of C3a was therefore not directed specifically at dividing cells.

To determine whether C3a was more active against transformed than primary fibroblasts, a comparison was made using MRC 5 and human adult skin fibroblasts as the primary lines, and HuS$^+$L$^-$ and MSV-3T3 as the transformed cells. 3T3 cells were considered partially transformed, since they did not show good contact inhibition. All the cell lines were grown in multiwell plates to give high, moderate and sparse cell numbers before treatment with C3a. The 3 cell concentrations all gave similar results, and the effect of C3a on moderate cell concentration is shown in Table IV. The primary human fibroblast lines were not lysed by concentrations of C3a that were cytolytic to both the transformed mouse and human cell lines and to 3T3.

DISCUSSION

The experiments with 3T3 fibroblasts growing at varying concentrations showed greater cytolysis on treatment with C3a when there was a moderate cell concentration, which implied that cells growing

TABLE III.—*Effect of quiescence on lysis on MSV-3T3 fibroblasts by C3a*

| C3a concentration ($\mu$g/ml) | Caffeine pretreatment: Nil $4 \times 10^5$/well | 500 $\mu$g/ml $3 \times 10^5$/well | 700 $\mu$g/ml $3 \times 10^5$/well | 900 $\mu$g/ml $2{\cdot}5 \times 10^5$/well |
|---|---|---|---|---|
| 10 | 69·0 (7·6) | 57·2 (17·0) | 65·4 (0·6) | 67·3 (0·6) |
| 5 | 40·9 (1·6) | 47·0 (0·2) | 52·2 (3·4) | 54·3 (2·6) |
| 2·5 | 29·5 (1·4) | 35·5 (1·8) | 31·3 (3·4) | 33·4 (1·0) |
| Nil | 15·9 (1·6) | 20·3 (0·2) | 23·4 (0·2) | 27·2 (2·0) |
| [$^{14}$C]TdR incorporation | 11638 (1922) | 1395 (50) | 657 (170) | 294 (20) |

Results are the mean of duplicate wells. The range is given in brackets.
Lysis is expressed as % loss of $^{51}$Cr.
0·02 $\mu$C[$^{14}$C]TdR was incubated with control cultures in 1 ml for 3 h.

TABLE IV.—*Lysis by C3a of various fibroblast lines*

| C3a concentration ($\mu$g/ml) | Human adult skin $1{\cdot}3 \times 10^5$/well | MRC 5 $9 \times 10^4$/well | HuS$^+$L$^-$ $9 \times 10^4$/well | 3T3 $5 \times 10^4$/well | MSV-3T3 $5 \times 10^4$/well |
|---|---|---|---|---|---|
| 15 | 44·6 (33·5–54·2) | 20·7 (16·8–24·6) | NT | NT | NT |
| 10 | 14·4 (12·5–15·9) | 24·5 (16·0–32·4) | 73·2 (72·1–74·3) | 69·9 (65·0–79·8) | 83·3 |
| 5 | 7·1 (6·2–7·9) | 12·9 (12·4–12·9) | 29·0 (28·3–29·7) | 46·8 (27·5–70·7) | 72·3 (67·3–77·3) |
| Nil | 5·8 (4·8–6·4) | 10·5 (10·1–11·1) | 12·7 (11·6–13·7) | 18·5 (11·5–22·6) | 23·0 (19·7–25·1) |

Lysis is expressed as % loss of $^{51}$Cr from the cells.
Results are the mean of triplicate cultures except for 3T3 which is the mean of 5 cultures and MSV-3T3 which is the mean of duplicate cultures.
The range is given in brackets.
NT: not tested.

optimally were more susceptible to C3a. However, when cells were pre-treated with caffeine, the results showed clearly that quiescent fibroblasts were as susceptible to C3a as rapidly dividing cells.

The comparison of several abnormal and primary cell lines showed that the primary human fibroblast lines MRC 5 and adult skin fibroblasts were more resistant to lysis by C3a than were MSV-transformed mouse fibroblasts and MSV-transformed human amnion cells. The human HuS$^+$L$^-$ cells did not produce virus, whereas the MSV-3T3 cells were producing virus at the time of the experiment. These experiments show that C3a is more cytolytic to abnormal than to normal cells. These findings are in agreement with those of Ferluga *et al.* (1976).

Many workers have shown that cell-surface changes are associated with transformation and malignancy (review article by Nicholson, 1976). Transformed cells agglutinate with lectins more readily than normal cells, show greater membrane motility and demonstrate new antigens on their surface, such as tumour-associated antigens, virus antigens and Forssman antigen. It is possible that C3a attacks *via* a site which is more available on transformed cells.

Earlier work by Lai A Fat & Van Furth (1975) and by Bentley *et al.* (1976) has demonstrated that macrophages can synthesize C3, and Schorlemmer & Allison (1976) showed that activated macrophages release enzymes capable of C3 cleavage. Ferluga *et al.* (1978) showed that activated macrophages killed tumour cells and liberated C3a into the medium. Since the present experiments have shown that C3a is more cytolytic to abnormal than to normal cells, this may prove important as a mechanism in tumour rejection.

Wuepper *et al.* (1972) showed that 20ng C3a caused an inflammatory reaction when injected into guinea-pig skin, so that it is unlikely that the concentrations of C3a used in the present experiments would be found circulating *in vivo*. However, it is possible that C3a generated by the action of macrophage enzymes on C3 could produce local concentrations of the order used in these experiments.

We would like to thank Dr H. U. Schorlemmer and Dr Bitter-Suermann, Institute of Medical Microbiology, University of Mainz, Germany, for kindly providing the C3a, Dr J. Harvey for supplying Hu S$^+$L$^-$, 3T3 and MSV-3T3 cells and Mr P. Narcissi for supplying the human adult fibroblasts.

### REFERENCES

Bentley, C., Bitter-Suermann, D., Hadding, U. & Brade, V. (1976) *In vitro* synthesis of factor B of the alternative pathway of complement activation by mouse peritoneal macrophages. *Eur. J. Immunol.*, **6**, 393.

Bitter-Suermann, D., Hadding, U., Melchert, F. & Wellensiek, H. J. (1970) Independent and consecutive action of the complement components C5, C6 and C7 in immune haemolysis. I: Preparation of EAC 1-5 with purified guinea pig C3 and C5. *Immunochemistry*, **7**, 955.

Bokisch, V. A. & Müller-Eberhard, H. J. (1970) Anaphylatoxin inactivator of human plasma: its isolation and characterization as a carboxypeptidase. *J. Clin. Invest.*, **49**, 2427.

Cochrane, C. G. & Müller-Eberhard, H. J. (1968) The derivation of two distinct anaphylatoxin activities from the third and fifth components of human complement. *J. Exp. Med.*, **127**, 371.

Dias da Silva, W., Eisele, J. W. & Lepow, I. H. (1967) Complement as a mediator of inflammation. III Purification of the activity with anaphylatoxin properties generated by interaction of the first four components of complement and its identification as a cleavage product of C′3. *J. Exp. Med.*, **126**, 1027.

Ferluga, J., Schorlemmer, H. U., Baptista, L. C. & Allison, A. C. (1976) Cytolytic effects of the complement cleavage product, C3a. *Br. J. Cancer*, **34**, 626.

Ferluga, J., Schorlemmer, H. U., Baptista, L. C. & Allison, A. C. (1978) Production of the complement cleavage product, C3a, by activated macrophages and its tumorolytic effects. *Clin. Exp. Immunol.*, **31**, 512.

Goodman, M., Weigle, W. O. & Hugli, T. E. (1980) Inability of the C3a anaphylatoxin to promote cellular lysis. *Nature*, **283**, 78.

Lai a Fat, R. F. & van Furth, R. (1975) *In vitro* synthesis of some complement components (C1q, C3 and C4) by lymphoid tissues and circulating leucocytes in man. *Immunology*, **28**, 359.

Nicholson, A., Brade, V., Schorlemmer, H. U., Burger, R., Bitter-Suermann, D. & Hadding, U. (1975) Interaction of C3b, B and D in the alternative pathway of complement activation. *J. Immunol.*, **115**, 1108.

Nicholson, G. L. (1976) Trans-membrane control of the receptors of normal and tumour cells. II: Surface changes associated with transformation and malignancy. *Biochim. Biophys. Acta*, **458**, 1.

Pardee, A. B. & James, L. J. (1975) Selective killing of transformed baby hamster kidney (BHK) cells. *Proc. Natl Acad. Sci., U.S.A.*, **72**, 4994.

Peebles, P. T., Fischinger, P. J., Bassin, R. H. & Papageorge, H. F. (1973) Isolation of human amnion cells transformed by rescuable murine sarcome virus. *Nature (New Biol.)*, **242**, 98.

Schorlemmer, H. U. & Allison, A. C. (1976). Effects of activated complement components on enzyme secretion by macrophages. *Immunology*, **31**, 781.

Schorlemmer, H. U., Davies, P. & Allison, A. C. (1976) Ability of activated complement components to induce lysosomal enzyme release from macrophages. *Nature*, **261**, 48.

Wuepper, K. D., Bokisch, V. A., Müller-Eberhard, H. J. & Stoughton, R. B. (1972) Cutaneous responses to human C3 anaphylatoxin in man. *Clin. Exp. Immunol.*, **11**, 13.